# Bio-hydrovoltaic technology: advancing from non-living to living hydrovoltaic systems

**DOI:** 10.1093/nsr/nwaf313

**Published:** 2025-08-04

**Authors:** Qichang Hu, Xiuyu Lin, Guoping Ren, Shungui Zhou

**Affiliations:** College of Resources and Environment, Fujian Agriculture and Forestry University, Fuzhou 350002, China; Fujian Key Laboratory of Agricultural Information Sensoring Technology, College of Mechanical and Electrical Engineering, Fujian Agriculture and Forestry University, Fuzhou 350002, China; Fujian Key Laboratory of Agricultural Information Sensoring Technology, College of Mechanical and Electrical Engineering, Fujian Agriculture and Forestry University, Fuzhou 350002, China; College of Resources and Environment, Fujian Agriculture and Forestry University, Fuzhou 350002, China; College of Resources and Environment, Fujian Agriculture and Forestry University, Fuzhou 350002, China

**Keywords:** bio-hydrovoltaic, hydrovoltaic effect, biomaterials, living hydrovoltaic, non-living hydrovoltaic

## Abstract

Bio-hydrovoltaic systems, which leverage the energy embedded in water's natural processes, present a promising avenue for sustainable energy production. Despite notable advances in non-living hydrovoltaic systems, research into biological materials remains relatively underdeveloped, leaving their unique advantages and long-term reliability potential largely untapped. This Review presents a comprehensive synthesis of progress in the field, with a particular focus on the evolution from non-living to living hydrovoltaic systems, and a precise articulation of their conceptual boundaries. We underscore a shift from structure-oriented material engineering toward biofunction-enabled energy conversion—a transition that enables breakthroughs in power output, system durability and environmental adaptability. Through systematic comparisons of mechanisms, biomaterial sources, performance metrics and emerging application scenarios, we identify current bottlenecks and propose targeted strategies. In light of recent trends, living hydrovoltaic systems hold particular promise as a forward-looking direction, offering a transformative platform for future hydrovoltaic internet, intelligence and ecological integration at scale.

## Teaser

This Review delineates the conceptual and material transition from non-living to living hydrovoltaic systems, establishing bio-hydrovoltaics as a frontier in sustainable energy conversion from water.

## INTRODUCTION

The global energy crisis presents one of the most critical challenges of our time, demanding sustainable solutions to replace dwindling fossil fuels while reducing environmental harm. The Earth's water cycle, a fundamental natural process encompassing evaporation, condensation and precipitation, harbors immense energy potential [1]. During this cycle, water molecules undergo multiple phase transitions, such as evaporation and condensation, each associated with energy absorption or release, predominantly manifesting as low-grade heat that remains largely untapped [2]. In this context, hydrovoltaic technologies have emerged as transformative tools for water-energy conversion, directly generating electricity through water and nanostructured material interactions such as moisture adsorption, evaporation and drag [3,4]. Unlike conventional power generation, these systems require no complex mechanical parts or external energy input, making them highly adaptable and versatile across environmental conditions.

Hydrovoltaic technology has evolved from an initial focus on traditional inorganic materials—such as carbon nanotubes and graphene—toward increasing interest in bio-systems. In 2014, studies demonstrated electricity generation through ion coupling as water flowed over graphene [5]. By 2017, stable power generation driven by natural water evaporation marked a milestone in practical applications [6]. The formal proposal of the ‘hydrovoltaic effect’ in 2018 provided theoretical foundations and catalyzed further hydrovoltaic-disciplinary development. As research has progressed, compared with the poor biocompatibility, difficulties in device maintenance and updating, and high costs of traditional hydrovoltaic materials, biomaterials have gained prominence due to their intrinsic hydrophilicity, biocompatibility and efficient water utilization, making them ideal candidates for bio-hydrovoltaic electricity generation (Bio-HEG) [7].

**Figure 1.**
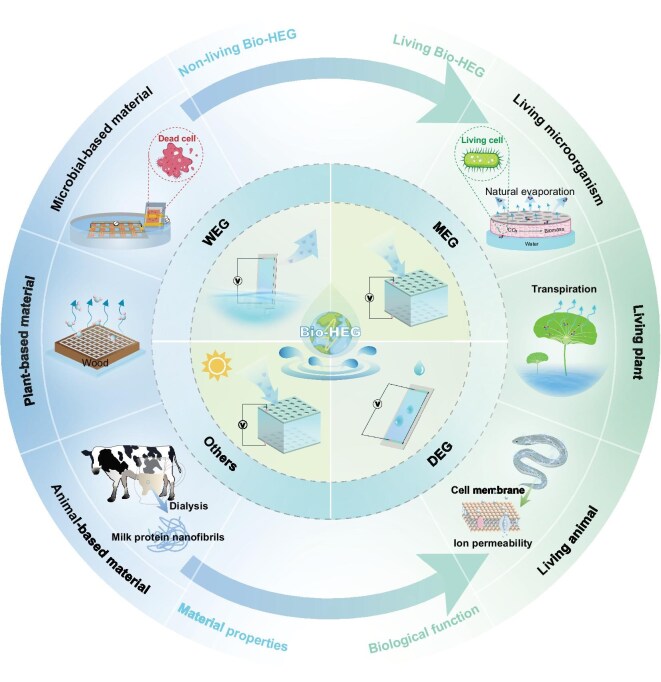
Development and diagram classification of the Bio-HEG [9,11–15].

**Figure 2.**
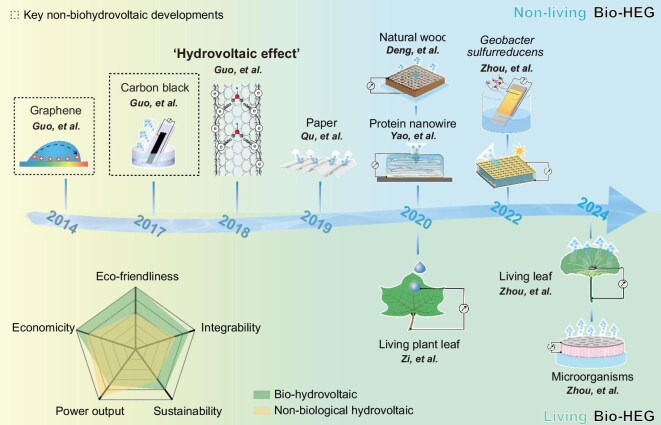
Timeline of Bio-HEG development [2,5,6,8–12,16–18].

This Review introduces a new classification of Bio-HEG technologies: non-living hydrovoltaics and living hydrovoltaics (Fig. 1). Non-living hydrovoltaics leverage static biological materials such as cellulose, wood and microbial protein nanowires, where energy harvesting primarily arises from spontaneous processes like water adsorption or evaporation. Their performance is closely tied to environmental stability. For instance, in 2019, a paper-based hygroelectricity generator achieved 0.24 V and 12 nA output under 70% relative humidity (RH) with excellent cycling stability [8]. In 2020, wood-based nanogenerators exploiting 3D microchannel structures significantly enhanced energy output [9]. Simultaneously, stable electricity generation using microbial protein nanowires further validated the potential of non-living materials [10] (Fig. 2). In contrast, living hydrovoltaic systems offer dynamic capabilities for water utilization and energy conversion, capitalizing on dynamic biological processes—such as microbial metabolism and plant transpiration—for more efficient hydrovoltaic energy conversion. It is important to distinguish this from bioinspired hydrovoltaics, which are inspired by the biological structures, functions or mechanisms found in nature and applied to hydrovoltaic technology, without using living organisms; instead, they primarily utilize synthetic or non-living biomaterials. Microorganisms transform water and nutrients into bioelectricity through metabolic activity and electron transport chains, while plants sustain energy cycling via transpiration [11,12]. The electrocyte-based energy generation in electric eels provides direct inspiration for bio-inspired hydrovoltaics [13]. These biological dynamics offer unique advantages in overcoming the limitations of static systems, marking a breakthrough in energy harvesting and conversion. Although living hydrovoltaics currently lag behind their non-living counterparts in overall performance, advances in synthetic biology, material science and device engineering are expected to unlock their full potential.

Recognizing the complementary attributes of non-living and living hydrovoltaic systems, we propose that the future of Bio-HEG lies in building multidimensional, multifunctional and intelligent Bio-HEG ecosystems. These systems transcend energy production, extending into environmental monitoring, healthcare and ecological integration. Living hydrovoltaic technology, as the core of this ecosystem, highlights its significant developmental potential in the field of future sustainable energy. While non-living hydrovoltaic materials contribute structural stability and predictable output, living hydrovoltaic systems introduce dynamic adaptability and self-healing properties. Their convergence may transform hydrovoltaic technologies from single-purpose devices into adaptive, ecologically synergistic platforms with capabilities such as intelligent sensing and autonomous regulation. With continued advances in living hydrovoltaics, these life-powered energy networks may evolve into self-sustaining infrastructures that respond dynamically to environmental changes, offering ultra-low maintenance and representing a cornerstone of future distributed energy architectures.

In light of the opportunities and challenges in the field of Bio-HEG, a systematic understanding of biomaterials is essential. This Review comprehensively synthesizes the latest developments (Table 1), beginning with definitions and distinctions among bio-hydrovoltaics, non-living hydrovoltaics and living hydrovoltaics. We delineate four representative working mechanisms—moisture-induced electricity generation (MEG), water evaporation-induced electricity generation (WEG), droplet-induced electricity generation (DEG) and other emerging forms—while highlighting current limitations in mechanistic understanding. Additionally, the Review systematically explores functional material sources across microbial, plant and animal kingdoms, offering a comparative analysis of performance characteristics and trade-offs between non-living and living Bio-HEG systems. Finally, this Review surveys emerging applications in energy harvesting, environmental sensing and health diagnostics, and provides a forward-looking perspective on methodological advances and future research directions essential for scaling bio-hydrovoltaic technologies.

**Table 1.** A compilation of Bio-HEG.

Mechanism	Materials	Current (µA)	Voltage (V)	Power density (µW·cm^−2^)	Condition	Ref.
Evaporation electricity generator	Wood	20	1	1.35	pH 13.4 NaOH	[19]
	Walnut shell	0.2 µA·mm^−2^	0.6	5.96	Deionized water	[20]
	Wood	320	1.1	6.75	2 M NaOH	[21]
	Paper	0.4	0.3		Water	[22]
	Paper	22	0.6		Deionized water	[23]
	Cellulose paper	48	0.41	0.022	0.6 M NaCl	[24]
	Carbon black particles/PVA@paper	6.0	1.2	0.25	Deionized water	[25]
	Carrot biochar	10	0.75	1.7	Deionized water	[26]
	Kelp biochar	49 µA·cm^−2^	0.34	1.88	Deionized water	[27]
	Natural leather	1	0.45	0.0678	Deionized water	[28]
	Rice husk biochar	0.04	0.16	0.366	55% RH	[29]
	Woody biochar	0.528	0.42	18.1 nW	Deionized water	[30]
	Textile	590.1	0.44	0.55 mW·cm^−3^	4 M NaCl	[31]
	Textile	1.75	0.35	110 nW	1 mM NaCl	[32]
	Cotton fabric	54.5	0.23	0.8 µW	0.6 M NaCl	[33]
	Mask strap	29.5	0.43		Deionized water	[34]
	Cotton fabric	3.91	0.53	255.5 nW	Dropping water	[35]
	Tencel fiber	0.6	0.73	0.168 µW	3.5 wt.% brine	[36]
	Starch-glass fibers	1.2	0.3	0.18	Deionized water	[37]
	Silk fibroin	0.13	4.82	0.139	Deionized water	[38]
	*G. sulfurreducens*	2.28	0.53	685.12	Deionized water	[17]
	Microorganisms*	0.87	0.34		Water	[12]
	Lotus leaf*	0.05	0.25		Water	[11]
	Microbial biofilms*	1.5	0.45	1	Deionized water	[14]
	Microalgae*	3.3	0.25		Water	[39]
	Green algae*	4.4	0.32		Deionized water	[40]
Moisture electricity generator	Leaf	49 µA·cm^−2^	0.5	12.43	74%–78% RH	[41]
	Balsa wood	77	0.57	0.71 µW·cm^−3^	85% RH	[42]
	Wood hydrogel	1.85	0.525		65% RH	[43]
	Wood	1200	0.65		35%–50% RH	[44]
	Print paper	0.015	0.25		70% RH	[8]
	Filter paper	5630	0.74		60% RH	[45]
	Cellulose paper	1.4	0.19		98% RH	[46]
	Cellulose sponge	477	0.47	74.73	45%–50% RH	[47]
	Textile	1.7	1	0.1	80% RH	[48]
	*Juncus effusus*	74.19	0.306	18.62 µW	60% RH	[49]
	Sodium alginate	4	0.25		70% RH	[50]
	Protein nanowires	0.25	0.50	4 mW·cm^−3^	50% RH	[10]
	*G. sulfurreducens* (ITO)	0.5	0.30	2.5	75% RH	[51]
	*G. sulfurreducens* (PET-ITO)	6.4	0.65	1.087	90% RH	[52]
	β-Lactoglobulin	2.9	0.65	38.88	90% RH	[15]
Droplet electricity generator	*G. sulfurreducens*	2	2.9	2.11	Water droplet	[53]
	Fabric		22.00	0.11 mW	Water droplet	[54]
	Paper		21.60	16 µW	Water droplet	[55]
	Resin	466	60.25		Water droplet	[56]
	Living plant leaves*	0.002	0.2	0.4 nW	Water droplet	[57]
	Living plant leaves*	4.1	1.3	1 µW	Water droplet	[16]
Other electricity generator	Fe_3_O_4_/wood	5.17	1	0.0743	Deionized water & light	[58]
	Natural wool	15 500	462	1 mW	5 cm & 4.5 Hz	[59]
	Textile	8	0.65		0.16 mL of deionized water	[60]
	Cellulose	69.7 A m⁻²	0.1401	257	0.5 M/0.01 M KCl	[61]
	Cellulose	13.7	0.94		Deionized water & light	[62]
	Cellulose and chitosan derivatives	0.5	0.186		Water & light	[63]
	Chitosan/carboxymethycellulose hydrogel	46	0.8	45.6	Artificial seawater & light	[64]
	Cellulon paper	7.5	0.78	0.7	25°C & 60% RH	[65]
	Cotton fabric	7.93	0.052	11.652	0.6 g h^−^^1^ brine	[66]
	Thermoelectric gelatin	∼0.7	6.4		55% RH & 290.1 K	[67]
	Bacterial cellulose	3.5	0.102	0.58 µW	0.5/0.01/0.5 M NaCl	[68]
	*G. sulfurreducens*	4 µA·cm^−^^2^	0.48	122.9	90% RH & light	[18]
	*G. sulfurreducens—*PSII	7.27.2	0.7	124	90% RH & light	[69]
	*Cladophora*	2.5	0.11	1010	0.5 M/0.01 M KCl	[70]
	Cyanobacterial*	0.57	0.52	0.017	90% RH & light	[71]

The materials marked with an asterisk (*) are living biomaterials.

## THEORETICAL FOUNDATIONS AND MECHANISMS OF BIO-HEG

### Fundamental principles and current understanding of different Bio-HEG

The electrical double layer (EDL) is a foundational physical phenomenon in the mechanism of hydrovoltaic electricity generation. When a solid surface comes into contact with an electrolyte solution, charge interactions induce the formation of two distinct layers: the first is the Stern layer, composed of ions (typically anions) directly adsorbed onto the solid interface; the second is the diffuse layer, where counterions are attracted by electrostatic forces but remain loosely distributed due to thermal motion, forming a gradient that decays with distance [3]. This interfacial charge distribution provides the essential electrochemical basis for hydrovoltaic power generation.

Whether in non-biological hydrovoltaics or bio-hydrovoltaics, the understanding of their power generation mechanism at the material level is derived from the development of the electrodynamic effect based on the EDL theory, which is related to ion movement. Therefore, in terms of material properties, their power generation performance is influenced by multiple factors, including pore structure, ionization level, conductivity and hydrophilicity. Bio-hydrovoltaic materials are richer in hydrophilic groups and benefit from natural porous structures, which help improve power generation performance. The stronger the hydrophilicity, the higher the dissociation, diffusion and conductivity of ions under the drive of water movement. Meanwhile, the porous structure provides channels for the evaporation of interior water molecules, which is a key factor for sustainable hygroelectricity by promoting the water cycle in the hydrovoltaic electricity generation (HEG). Additionally, from an energy perspective, bio-hydrovoltaic technology exhibits more complex characteristics. Unlike the static properties of inorganic materials, living biomaterials possess dynamic regulation capabilities. Beyond the simple electrodynamic mechanism, they also include contributions from biological metabolism, which collectively promote power output.

**Figure 3.**
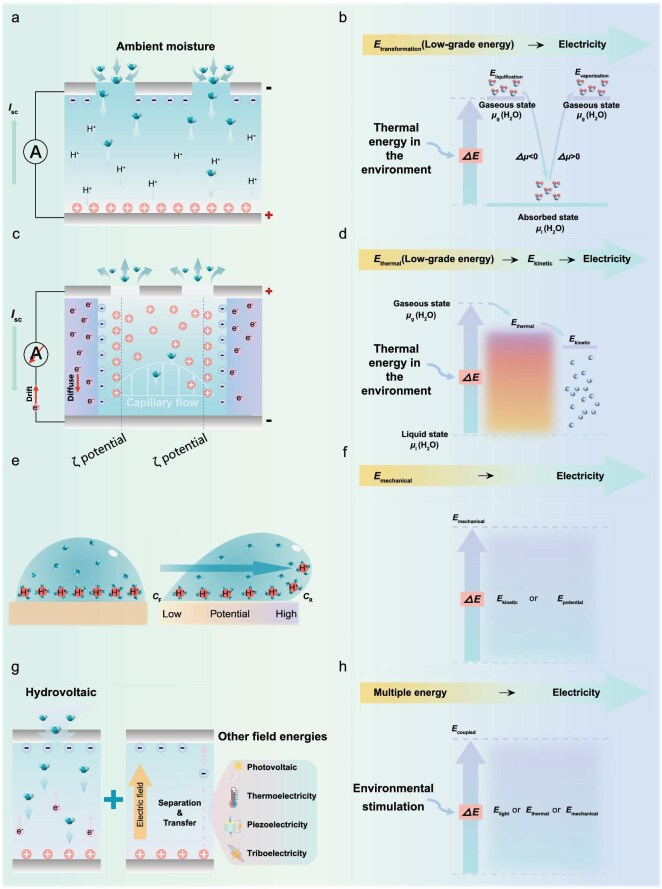
Various types of Bio-HEG according to different working mechanisms. (a) Charge transport mechanism of the MEG; (b) energy conversion schematic of the MEG; (c) charge transport mechanism of the WEG; (d) energy conversion schematic of the WEG; (e) charge transport mechanism of the DEG; (f) energy conversion schematic of the DEG; (g) charge transport mechanism of other types of generators; (h) energy conversion schematic of other types of generator.

A refined understanding of EDL behavior has revealed that the charge dynamics at the solid–liquid interface play a critical role in energy conversion processes. Depending on the type of water movement involved, Bio-HEG can be categorized into several working mechanisms. In this review, we examine these mechanisms in detail from the dual perspectives of mass balance (ion transport) and energy conservation (energy transfer), as illustrated in Fig. 3.

#### Moisture-induced electricity generation

Conventional hydroelectricity generation typically relies on liquid water as a working medium, whereas MEG breaks through this limitation by harvesting vapor from the ambient water vapor, enabling energy conversion. MEG, as one of the core technologies in hydrovoltaic technology, has become a vital breakthrough direction in the field due to its unique advantages. First reported by Qu *et al*. in 2015, MEG was observed in graphene oxide films and attributed to an ion diffusion mechanism driven by a moisture-induced gradient. The fundamental mechanism involves the dynamic adsorption and desorption of water molecules, which induces an imbalance in surface charge distribution and drives directional electron flow [72].

The energy source of MEG stems from dynamic adsorption–desorption interactions between water molecules and nanoporous materials. When water vapor is adsorbed onto the surface functional groups of materials, chemical bonds or van der Waals interactions form, releasing substantial heat, a process that generates mobile surface charges. Conversely, the desorption process involves bond breakage, producing an endothermic effect. From an energy conservation perspective, the dynamic phase transition between water vapor (high enthalpy/high entropy state) and adsorbed water molecules (low enthalpy/low entropy state) drives energy generation in MEG (with enthalpy change around ∼2500 kJ·kg^−1^ and entropy change around ∼8 kJ·kg^−1^·K^−1^) [73]. From the perspective of ionic charge balance, the adsorption and desorption interactions of water molecules and solid surface charges establish a spontaneous charge gradient accompanying humidity gradient, driving electrical energy output (Fig. 3a).

Based on these mechanisms, MEG technologies have evolved along three main directions. Initially, Qu *et al.* employed gradient graphene oxide films (g-GOFs) through moisture/electric annealing (MeA), with its core mechanism leveraging gradient materials and asymmetric chemical structures [72]. By constructing a gradient distribution of water-absorbing functional groups (e.g. hydroxyl and carboxyl groups), hydrogen ions (H^+^) are induced to generate a concentration gradient, driving current production. This method involves complex processes and is challenging to scale up. Subsequently, researchers shifted towards asymmetric electrode design on uniform materials, with the core mechanism triggering regional solvation effects through electrode asymmetry, releasing ions (such as H⁺, Cl⁻) and forming charge separation. This method relies on electrodes, and output performance is constrained by material limitations. In recent years, Qu *et al.* developed a bilayer polyelectrolyte film where, after water molecule adsorption, the anion and cation membranes separately release Cl⁻/H⁺, forming an ionic concentration difference. This device eliminates the need for complex gradient design, relying solely on the inherent ionic characteristics of materials [74]. However, most MEG mechanisms based on these principles depend on humidity changes (ΔRH ∼30%) to trigger proton diffusion and cannot self-sustain gradients. Once the thin film becomes saturated with water adsorption, the concentration gradient disappears, causing the voltage to drop precipitously. Additionally, the large porous structure of these materials prevents the generation of stable humidity gradients, which are the primary reasons for their intermittent electricity generation.

To overcome these challenges, Yao *et al.* pioneered continuous electricity generation by fabricating a MEG device using protein nanowires extracted from *Geobacter sulfurreducens* (*G. sulfurreducens*) [10]. These microbial nanowires exhibit high electrical conductivity and intrinsic nanoscale porosity, supporting continuous water vapor adsorption and stable humidity gradients. Their microstructural characteristics, including electrical conductivity, hydrophilicity and porous structure, enhance moisture adsorption and ion conduction, demonstrating significant MEG performance. However, the complex experimental steps of nanowire extraction have substantially limited the material's practical applications.

Inspired by this work, Zhou *et al*. developed a series of MEG devices based on microbial biofilm materials, by utilizing whole-cell biofilms and biomimetic strategies to resolve the issues of unsustainable electricity generation and complex processing in traditional MEG devices [18,51,52,69,75]. For instance, through research on whole-cell *G. sulfurreducens* MEG, they verified the self-sustaining humidity gradient of microbial biofilm [51]. They further explored the interactions between hydrophilic groups, ion dissociation, porous structure and environmental factors during dynamic adsorption–desorption processes [75] (Fig. 3b).

#### Water-evaporation-induced electricity generation

In 1859, Quincke discovered the inverse effect of electroosmosis, now known as streaming potential, wherein electrolytes generate an electrical potential due to ion accumulation in the EDL when flowing through narrow channels under a pressure gradient [76]. In 2017, Guo and Zhou *et al.* first demonstrated a groundbreaking research result, directly harvesting energy from water evaporation using nanoscale carbon materials, marking a significant milestone in WEG [6].

Specifically, the water evaporation process is a natural phase transition that involves water molecules transforming from liquid to gaseous state by absorbing environmental heat. During this process, external water molecules enter charged nanochannels driven by a pressure gradient, forming a continuous water flow. Within these nanochannels, coulombic forces cause ions with opposite charges to the channel surface to form an EDL at the solid–liquid interface. Some ions move directionally with the water flow, creating an ionic concentration difference within the nanochannels and consequently generating a potential difference (streaming potential). Throughout this process, the effective conversion of kinetic energy to electrical energy is achieved through ion migration and heat transfer (Fig. 3c and d). The induced streaming potential (*V*_str_) and current (*I*_str_) can be expressed as follows [3,77]:


\begin{eqnarray*}
{{\mathrm{V}}}_{{\mathrm{str}}} &=& \frac{{{{\mathrm{\varepsilon }}}_{\mathrm{r}}{{\mathrm{\varepsilon }}}_0{\mathrm{\zeta \Delta P}}}}{{{\mathrm{\eta \sigma }}}}, \\
{{\mathrm{I}}}_{{\mathrm{str}}} &=& {\mathrm{A\sigma }}\frac{{{{\mathrm{V}}}_{{\mathrm{str}}}}}{{\mathrm{l}}}= \frac{{{\mathrm{A}}{{\mathrm{\varepsilon }}}_{\mathrm{r}}{{\mathrm{\varepsilon }}}_0}}{{{\mathrm{\eta l}}}}{\mathrm{\zeta \Delta P}},
\end{eqnarray*}

where *ε_r_ε_0_, η* and *σ* represent the relative permittivity, viscosity and specific conductivity of liquid, respectively, and *A, ζ, ΔP* and *l* denote the cross-section area, zeta potential, pressure difference and pore length, respectively.

Early research primarily attributed the mechanism of evaporation-induced electricity generation to classical streaming potential, without fully considering the complex interactions between water molecules and material interfaces. In 2022, Guo *et al.* introduced the concept of ‘evaporation potential’, supplementing its role in maintaining continuous electricity generation through evaporation [78]. Moreover, while streaming potential theory effectively explains voltage generation, it cannot fully elucidate the mechanism of sustained current output. Inspired by previous research [14,79], Zhou *et al.* introduced an electron image model that effectively explains the mechanism of continuous current generation in WEG [11].

Biomaterials demonstrate exceptional performance in this context, owing to their abundant hydrophilic functional groups and hierarchical porous structures, which facilitate water adsorption, dissociation and ion transport, forming hydrovoltaic electric fields. These characteristics enable efficient energy conversion by utilizing environmental thermal energy and evaporation latent heat. Notably, both Zhou *et al.* and Yao *et al.* subsequently reported unprecedented evaporation-induced electricity generation phenomena in microbial biofilms, establishing prototypes of bio-WEG [14,17]. These discoveries laid the foundation for advancing hydrovoltaic technology using biological materials.

Recent research has explored the biological functionality of living materials for WEG. For instance, in 2024, Ren *et al*. demonstrated that microbial extracellular electron transfer could utilize hydrovoltaic electrons to sustain biofilm growth, strongly dependent on carbon fixation and nitrate reduction [12]. Similarly, Zhou *et al.*, inspired by natural plant transpiration, captured environmental latent heat through transpiration processes in living plant leaves, achieving sustained electrical power generation. These breakthroughs provide new perspectives for advancing bio-hydrovoltaic technologies [11].

### Droplet-induced electricity generation

A defining feature of hydrovoltaic technology is the coupling between mechanical motion and electrical output at the water–solid interface, as seen in dynamic processes such as raindrops, waves and water flows. When a charged surface comes into contact with water or an ionic solution, counter-ions form an exponentially decaying electric potential field within the EDL [4]. The dynamic movement of ions in the EDL stimulates electrons in the substrate or electrode [5,80,81]. Concurrently, the advancing front of the droplet induces pseudocapacitive charging, while the receding edge promotes discharging, as illustrated in Fig. 3e. In this process, the mechanical energy from the flow of ionic droplets on the device surface is converted into electrical energy (Fig. 3f) [5]. The energy conversion process of DEG is very rapid and represents a typical form of transient energy harvesting. The rolling of droplets on the material surface not only plays a role in transferring mechanical energy but also generates excess charge through the reallocation of electrons in the material's surface layer. Compared to other mechanisms, DEG can produce higher peak instantaneous voltages, a characteristic that is particularly important for applications in dynamic environments. Although various electrodynamic phenomena based on EDL theory have been studied, these typically generate ionic currents in liquids rather than the electronic currents required for power transmission, thus limiting practical applications.

In 2014, Guo *et al.* discovered that the movement of droplets on a monolayer of graphene generates millivolt-level electrical signals, referred to as ‘drawing potential’ [5]. This phenomenon couples the movement of ions in water with the redistribution of electrons in graphene, directly converting the water's kinetic energy into electrical energy. Since then, the mechanism of DEG has been developed, particularly in the configuration of devices utilizing sliding or falling droplets, which involves pseudocapacitance charging and discharging as droplets roll on the material surface, converting mechanical energy into electrical energy [77,82]. Meanwhile, by optimizing charge separation during the droplet impact process, the energy conversion efficiency has been improved [56,83]. Biomaterials, with their rich functional groups and high intrinsic conductivity, provide abundant active sites for stable EDL formation and efficient electron conduction [15,19,75]. Flexible and multifunctional biomaterials, including protein films and microbial biofilms, demonstrate strong adaptability to dynamic environments, achieving efficient mechanical–electrical energy conversion under complex, moisture-rich scenarios [53].

#### Other mechanisms

Beyond primary mechanisms, several additional hydrovoltaic-related energy harvesting strategies have been explored. For example, the osmotic pressure difference between river water and seawater represents a highly promising renewable energy source. The concept of generating electricity from this differential was first proposed by Loeb in 1975 [84]. With advancements in nanotechnology and membrane materials, the utilization of osmotic energy (or salinity gradient energy) has gained widespread attention and significant progress. Similarly, ocean waves represent another powerful energy source. For instance, a landmark paper by Stephen Salter from The University of Edinburgh, published in *Nature*, drew international attention to wave energy [85]. Salter introduced a design for efficiently harvesting energy from sea waves using specially shaped vanes, emphasizing the potential of wave power as a clean, sustainable and robust solution for future energy needs.

To further enhance the efficiency and adaptability of hydrovoltaic systems, researchers have increasingly focused on hybrid strategies that couple hydrovoltaic energy conversion with other mechanisms (Fig. 3g and h). The photovoltaic effect involves the utilization of light to excite electron–hole pairs in semiconductors, with an internal electric field promoting charge separation and current generation. In contrast, hydrovoltaic electricity generation typically relies on hydrophilic materials, which facilitate charge generation and transport through water absorption and diffusion. Biomaterials, as a class of highly hydrophilic substances, offer advantages such as excellent moisture absorption, biodegradability and environmental compatibility. For instance, Zhou *et al.* developed a self-assembled heterojunction composed of microbial biofilms to achieve a hybrid hydrovoltaic–photovoltaic generator (HPG) [18,69]. In a humid environment, water molecules facilitate the separation of photogenerated electrons and holes, reducing the conductivity of the films and decreasing the resistance to charge transfer and diffusion. Additionally, the directions of the photovoltaic and hydrovoltaic electric fields are aligned, and the coordination of the two fields results in a higher superimposed electric field or voltage, thereby enhancing the overall energy conversion efficiency. Similarly, systems utilizing natural cyanobacteria like *Synechocystis* sp. PCC 6803 harness photosynthesis and moisture capture during the day and generate hydrovoltaic power at night [71]. Such designs also encompass coupling hydrovoltaics with piezoelectric [86], triboelectric [87] or thermoelectric mechanisms [67], which enables synergistic energy harvesting through biohybrids, biomimetics and thermal/moisture gradients.

### Limitations of current theoretical understanding

Based on current research progress, bio-hydrovoltaic technology faces significant theoretical challenges—particularly in understanding the mechanisms of dynamic hydrovoltaic energy conversion processes in living biological systems. Traditional studies of non-living hydrovoltaics primarily rely on static interface models [72,88], which, while effectively describing charge separation and transport on solid material surfaces, struggle to accurately capture the complex dynamic characteristics present in living bio-hydrovoltaic systems. Energy conversion within biological systems is a highly coupled, real-time regulated complex process involving multi-level cooperative mechanisms from molecules to cells, and ultimately to entire organisms. This process encompasses multidimensional dynamic mechanisms [11–13], for example, lotus leaves regulate the rate of transpiration and energy output in real time by controlling the opening and closing of stomata. This process involves the rapid response of intracellular signaling pathways and the dynamic regulation of ion channels. The opening and closing of stomata not only respond to changes in environmental temperature, humidity and light but also reflect the close relationship between molecular-level regulatory mechanisms and cellular activities. This allows the entire plant to effectively carry out gas exchange and moisture regulation under rapidly changing environmental conditions, ensuring a continuous supply of energy. Similarly, this highly coupled and dynamic regulatory characteristic is also evident in electroactive microorganisms (EAMs). The electron transfer reactions and metabolic pathways of EAMs occur within the cells, involving multiple essential molecular mechanisms, such as changes in enzyme activity and the dynamic regulation of signaling pathways. Environmental condition changes, such as the abundance of electron donors, pH levels and temperature, instantaneously affect the activity of ion channels in the cell membrane, thereby altering the concentration and potential of ions within the cells. This change further regulates the metabolic state of an EAM, enabling rapid responses of the organism to external signals while affecting the overall energy conversion efficiency of the living hydrovoltaic devices. These biological systems exhibit self-organization and adaptive abilities that surpass traditional physical models. However, the current lack of a comprehensive theoretical framework capable of fully describing these dynamic processes hinders our understanding of bio-hydrovoltaic mechanisms and affects the transition of this technology from fundamental research to practical applications.

Future research must transcend traditional static models and develop a dynamic, cross-scale theoretical paradigm that describes dynamic energy conversion in biological systems to bridge the gap in our understanding. Developing such a paradigm requires the convergence of multiple disciplines, including biology, physics, electrochemistry and materials science, to delve into the essential mechanisms of energy conversion in biological contexts. Specific research directions should include the development of modeling and simulation for dynamic processes, investigations of multi-scale coupling mechanisms, and analysis of complex interfacial interactions. Only through this interdisciplinary, multi-scale approach can we uncover the technical potential of bio-hydrovoltaic technologies and lay a solid theoretical foundation for the development of future green energy technologies.

## RESEARCH ADVANCES IN BIO-HYDROVOLTAIC MATERIALS

Bio-hydrovoltaic materials are functional materials prepared using biological resources from nature. Based on their sources and characteristics, bio-hydrovoltaic materials can be categorized into three main types: microbial-based, plant-based and animal-based materials. Microbial-based materials, such as bacteria and algae, possess self-healing capabilities, strong environmental adaptability and the potential for genetic engineering modification. Their natural origin endows them with environmental friendliness and controllable production costs. Plant-based materials feature a natural gradient in pore size distribution and oriented transport channels, facilitating moisture transport and energy conversion, while being low cost and abundant in resources. Animal-based materials demonstrate excellent biocompatibility and mechanical properties, showcasing significant application potential. Despite their different sources, these biomaterials share common characteristics: compared to traditional inorganic hydrovoltaic materials, they typically have richer hydrophilic groups (carboxyl, hydroxyl, amino) and favorable porous structures that collectively enhance moisture and ion transport as well as energy conversion. Current research focuses on utilizing the unique structural and functional properties of these biomaterials to develop efficient and stable hydrovoltaic power generation devices. This section will provide a detailed overview of the current research advancements in bio-hydrovoltaic materials.

### Microbial-based hydrovoltaic materials

**Figure 4.**
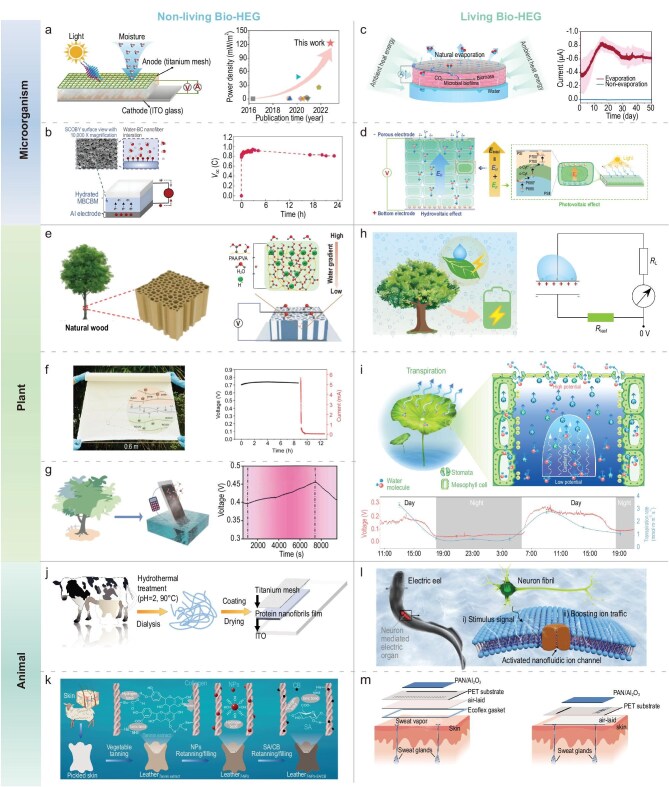
Overview of non-living and living Bio-HEG. (a) Mechanism of hydrovoltaic and photovoltaic energy generation in *G. sulfurreducens–*CN*_x_* HPGs [18]. Copyright 2023, Elsevier. (b) Structure and working principle of a hydrovoltaic device based on MBCBM [89]. Copyright 2023, Elsevier. (c) Diagram of Bio-HEG utilizing an electroactive microbial biofilm [12]. Copyright 2024, Springer Nature. (d) Schematic illustration of a cyanobacteria-based HPG [71]. (e) Hydrovoltaic power generation mechanism of wood-based hydrogels [43]. Copyright 2024, American Chemical Society. (f) Power generation performance and mechanism of dual-layer polyelectrolyte-functionalized paper conductors [45]. Copyright 2024, American Chemical Society. (g) WEG based on WBC [30]. Copyright 2023, Elsevier. (h) Fully biodegradable DEG based on leaves of living plants and circuit diagram [16]. (i) Principle of transpiration power generation in the LTG system and long-term electricity generation test results [11]. Copyright 2024, Springer Nature. (j) Bio-HEG utilizing protein nanofibrils from β-lactoglobulin [15]. (k) WEG device based on natural bio-leather [28]. Copyright 2024, Elsevier. (l) Electricity generation through ion gradients across electrocytes in electric eels [13]. Copyright 2023, Wiley-VCH GmbH. (m) Bio-HEG using natural leather [91]. ITO, indium tin oxide; SCOBY, symbiotic culture of bacteria and yeast; BC bacterial cellulose; *R*_L_, load resistance; *R*_leaf_, leaf resistance; NP, nanoparticle; T-NPs, vegetable-tanned leather re-tanned/filled with nanoparticles; CB, charcoal black; SA, sodium alginate; PAN, polyacrylonitrile.

Microorganisms, including bacteria, fungi and algae, exhibit remarkable biodiversity and unique biochemical characteristics, providing rich biomass sources for novel Bio-HEGs. Initial research in this domain predominantly focused on non-living hydrovoltaics, primarily utilizing microbial structural components and metabolic products to construct hydrovoltaic functional materials. For instance, in 2020, Yao *et al.* prepared a MEG device using *G. sulfurreducens* nanowire films. This research revealed that self-maintained humidity gradients are crucial for achieving continuous electricity generation, while densely arranged nanopores provide critical pathways for charge transport and separation [10]. In the field of EAMs, *G. sulfurreducens*—a typical bacterium capable of secreting protein nanowires—established the foundational basis for Bio-HEG development. However, the purification of nanowires for hydrovoltaic device fabrication not only involves complex processes but also suffers from initial voltage decay and limited output voltage. Consequently, researchers began simplifying material preparation techniques and modulating surface hydrophilicity and heterojunction design to enhance overall hydrovoltaic device performance [17,18,51] (Fig. 4a). In 2023, another research team developed a metal-coated bacterial cellulose nanofibrous bilayer membrane (MBCBM) (Fig. 4b), significantly enhancing electrical output by introducing Schottky barriers, achieving a maximum voltage of 0.935 V and current of 7.51 mA. Future research will focus on optimizing layer-coating technologies and improving environmental adaptability [89].

As research progresses, researchers have begun directly leveraging the physiological metabolism and electron transfer capabilities of living microorganisms to support hydrovoltaic electricity generation. A research team from Amrita Engineering Institute in India (2023) developed a Bio-HEG device based on freshwater green algae (*Pithophora roettleri*), demonstrating the immense potential of microbial surface charges in electricity generation, and pioneered a new approach for hydrovoltaic technologies using living biomaterials [40]. In 2024, Zhou *et al.* investigated the growth mechanisms of EAMs driven by hydrovoltaic energy [12]. Results showed that *Rhodopseudomonas palustris* achieved carbon fixation and nitrate reduction through extracellular electron transfer. These microorganisms utilized hydrovoltaic electrons induced by water evaporation, generating a stable voltage of 0.34 V and a short-circuit current of 0.87 µA, supporting microbial growth in oligotrophic environments. This discovery challenges the long-standing perception that microorganisms can only use chemical and light energy, proposing hydrovoltaic energy as a third novel pathway for microbial energy utilization (Fig. 4c). Subsequently, the team proposed an HPG based on cyanobacteria that fully utilizes the intrinsic photovoltaic and hydrovoltaic effects of cyanobacteria, achieving all-day stable power output [71]. Moreover, the research further analyzed the growth adaptability and humidity responsiveness of cyanobacteria under different environmental conditions, providing a theoretical basis for constructing novel green energy devices (Fig. 4d).

Microbial-based hydrovoltaic materials have made significant advances in energy conversion efficiency and multifunctional integration. However, challenges remain, including complex preparation processes, high environmental dependence and high scale-up costs. Future research can further enhance performance by regulating microbial metabolism through genetic engineering, developing biomimetic heterojunction structures and optimizing device packaging processes, thereby promoting their specialized applications in flexible electronics and energy supply in extreme environments.

### Plant-based hydrovoltaic materials

Plant-based materials possess unique molecular structures and rich functional groups, primarily derived from natural biopolymers such as cellulose and lignin. These materials are characterized by their widespread availability, low cost and environmental friendliness. Currently, plant-based Bio-HEG represents the most extensively researched and diverse category within bio-hydrovoltaic technologies. Most studies have focused on non-living plant-based hydrovoltaic materials, particularly lignocellulosic materials (such as wood), cellulose-based materials (such as paper) and biomass-derived carbon materials. However, in recent years, research on living plant-based materials has gradually gained attention. Living materials rely on natural physiological activities of plants (such as transpiration) for energy conversion, enabling spontaneous energy harvesting without external driving forces. The following section will provide a detailed exposition of plant-based Bio-HEG.

#### Non-living plant-based hydrovoltaic materials

Natural wood demonstrates outstanding performance in Bio-HEG due to its excellent hydrophilicity, porosity and anisotropic structure. The inherent porous structure and abundant polar functional groups in wood effectively promote water absorption and ion migration, achieving efficient electrical energy output [19,42]. In recent years, nanoscale engineering modifications of wood (such as delignification and chemical treatments) have significantly enhanced its ionic conductivity and surface charge density. For instance, Zhang *et al.* developed a wood hydrogel-based MEG (Fig. 4e), which achieved 0.525 V (*V*_oc_) and 1.85 µA (*I*_sc_) by impregnating polyvinyl alcohol (PVA) and polyacrylic acid (PAA) hydrogels into delignified wood [43]. These modification techniques preserve wood's inherent mechanical strength and environmental friendliness, revealing tremendous application potential in green energy domains.

Many research efforts in plant-based Bio-HEG have utilized paper as a core functional layer. Paper, derived from plant sources, is an environmentally friendly, easily accessible and biodegradable material. Most papers retain natural capillary structures and rich hydrophilic functional groups similar to wood, efficiently absorbing and transporting environmental moisture, providing ideal channels for ion migration. In 2019, Qu *et al.* prepared a MEG using an untreated printing paper (1.5 cm^2^ area) that could generate 0.25 V (*V*_oc_) and 15 nA (*I*_sc_) [8]. While the authors demonstrated linear power output growth through simple series and parallel configurations, the performance improvement for individual devices remained limited. Consequently, researchers optimized paper-based device performance by introducing functionalized coatings or multi-layer composite structures. For example, Liu *et al.* developed a MEG based on a bilayer polyelectrolyte functionalized paper conductor. By coating a polyelectrolyte layer on the cellulose network, they significantly improved ion dissociation and migration efficiency (Fig. 4f) [45].

In recent years, Bio-HEG using biochar materials has garnered significant attention. Woody Biochar (WBC) represents one of the most typical biochar materials, characterized by its porous structure, high specific surface area and abundant surface polar functional groups, with widespread sources and low preparation costs. Tian *et al.* prepared WBC through pyrolysis of lignocellulosic raw materials (Fig. 4g). Benefiting from the rich hydrophilic functional groups and micro-nanoscale porous structures, the device could achieve stable electrical energy output without additional hydrophilic treatment [30]. A single device could generate 0.42 V (*V*_oc_) and 528 nA (*I*_sc_), with series connection enabling a 7 V voltage output, suggesting potential power supply for small electronic devices.

Compared to WBC, other plant-based biochar materials—such as seaweed and fruit/vegetable waste—also demonstrate exceptional hydrovoltaic electricity generation capabilities after carbonization. For instance, carbonized carrot slices possess a natural 3D porous structure, with high zeta potential and excellent surface hydrophilicity forming the foundation for superior electrical energy output. A WEG device constructed from carbonized carrot slices could achieve an open-circuit voltage of 0.8 V and a maximum power density of 1.7 µW·cm^−2^ [26]. Similarly, carbonized seaweed materials have emerged as high-quality candidates for Bio-HEG, leveraging their rich surface functional groups and enhanced hydrophilicity. These biochar materials from diverse sources can further improve conductivity and interfacial electrochemical activity through optimized carbonization processes and nanostructure design, enhancing hydrovoltaic performance [27].

#### Living plant-based hydrovoltaic materials

Living plants offer an entirely novel research direction for Bio-HEG, leveraging their unique physiological functions and self-maintenance capabilities. Unlike non-living plant materials, living plants not only possess inherent physicochemical properties but also continuously engage in natural physiological metabolic activities during hydrovoltaic energy conversion, including transpiration, water and ion transport, and photosynthesis. This electricity generation mode, coupled with plant metabolic activity, enables bio-hydrovoltaic energy conversion that is not solely dependent on external environmental moisture conditions, thereby achieving spontaneous hydrovoltaic energy transformation with the advantages of environmental friendliness, self-healing and long-term stable operation. For instance, researchers discovered that when electrodes are implanted in plant stems, living plants generate electrical signals during transpiration, with inorganic salt additions significantly enhancing voltage. This power generation phenomenon originates from charge separation in the xylem during plant transpiration [90]. Wang *et al.* investigated a fully biodegradable DEG that utilizes living plant leaves to collect environmental energy, effectively harvesting electrical energy from electrostatic and mechanical energy generated by water droplet impacts. By selecting five different plant leaves as experimental subjects, they demonstrated the method's broad applicability (Fig. 4h) [16]. In 2024, Zhou *et al.* developed an innovative living lotus leaf transpiration generator (LTG), directly capturing environmental latent heat through leaf transpiration to achieve continuous power generation (Fig. 4i) [11]. Research indicated that the LTG could generate 0.25 V (*V*_oc_) and 50 nA (*I*_sc_) under natural conditions, with further output amplification possible through series or parallel connections. Compared to traditional artificial hydrovoltaic devices, the LTG fully leverages the natural transpiration processes of plants without additional water source requirements, showcasing the immense potential of plants in green energy technologies.

It is worth noting that non-living and living plant-based hydrovoltaic materials each possess unique advantages. The former is more amenable to material modification and optimization to enhance power generation performance, while the latter achieves more green and sustainable energy collection through plant self-maintenance capabilities. Future research should focus on further exploring and combining the characteristics of these two material types to achieve more efficient energy conversion in the Bio-HEG domain.

### Animal-based hydrovoltaic materials

Compared to microbial and plant-based materials, animal-based hydrovoltaic materials have been less extensively researched. Animal-based hydrovoltaic materials originate from animals or their derivatives, typically characterized by excellent mechanical properties, rich functional groups and high biocompatibility. These materials perform energy conversion through inherent physical and chemical properties, either utilizing non-biologically active animal physiological metabolism or relying on biological activities in living materials, thereby introducing new possibilities for sustainable energy technologies.

Protein nanofiber films represent a crucial non-living animal-based material. For instance, nanofiber films prepared from β-lactoglobulin in milk possess high specific surface areas and abundant surface functional groups. Yuan *et al.* constructed a Bio-HEG device using these fibers, with the electricity generation mechanism primarily dependent on water molecule adsorption and diffusion on the fiber surface [15]. Under 90% RH, the device achieved an open-circuit voltage of 0.65 V and a short-circuit current of 2.9 mA, demonstrating the broad prospects of milk-derived biomaterials in hydrovoltaic technology (Fig. 4j). Natural animal leather, primarily composed of collagen fibers, features multi-level microscopic structures and rich functional groups that provide excellent moisture permeability, facilitating water evaporation and transport. Chemical modification can enhance leather's hydrophilicity, improving solid–liquid interface wettability and maintaining energy output. Guan *et al.* developed a WEG device using tanned sheepskin, incorporating plant extracts, nanoparticles and carbon black as functional fillers through tanning and re-tanning processes. When six devices were connected in series, the output voltage reached 3 V, maintaining stable electrical output under various environmental conditions (Fig. 4k) [28]. Due to their exceptional thermal stability, leaching resistance, mechanical performance, biocompatibility and low cost, leather-based WEG devices offer extended use life, convenient storage and transportation, and promising application prospects. Beyond leather and protein nanofibers, other animal-based materials (such as silk and animal bone glue) have also undergone preliminary exploration in hydrovoltaic technologies. Silk, for instance, has been used to prepare flexible hydrovoltaic devices due to its high strength and good conductivity, while animal bone glue, rich in carboxyl and amino functional groups, is suitable for interface modification and energy conversion [38,67].

Research on living animal-based hydrovoltaic systems remains extremely limited, primarily focusing on utilizing animal skin sweat glands and combining them with other hydrovoltaic materials to achieve energy conversion through metabolic activities, demonstrating self-maintenance and environmental adaptability. For instance, electric eels possess unique ‘electric organs’ composed of thousands of electroplates arranged in parallel, capable of generating voltages up to 600 V and currents of 1 A. Their energy generation depends on directional ion channel regulation and charge accumulation, enabling rapid and efficient energy output (Fig. 4l). Zhang *et al.* assembled a 3D gel electric motor using layer-frozen-dried nanofluidic cable fibers, mimicking this mechanism and achieving a peak power density of 23 W·m^−2^, outperforming traditional membranes [13]. Furthermore, Zheng *et al.* developed a wearable hydrovoltaic device based on human skin (Fig. 4m) [91]. This device integrated power generation, sweat ion detection and Bluetooth wireless signal transmission. The system demonstrated the correlation between sweat Cl^−^ concentration and secretion rate, providing insights for preventing liquid–ion imbalance. During long-term operation, the device exhibited robust performance, highlighting its potential for wearable electronic applications.

Although living animal-based hydrovoltaic research remains constrained by medical ethics and interdisciplinary complexities, it is crucial to recognize that animals (including humans) are composed of 50%–70% water. Within this context, many internal biological flows—including blood, tissue fluids and ions—potentially generate corresponding hydrovoltaic effects. These signals may potentially influence internal biological activities, with hydrovoltaic output comparable to neuronal signal magnitudes. Developing living animal-based hydrovoltaic technologies that evolve from static physicochemical characteristics to dynamic biological functional drivers holds significant importance. Such development not only expands energy collection pathways but also provides novel insights for innovative interdisciplinary biological technologies.

## APPLICATIONS OF BIO-HEG

Bio-HEG leverages the energy embedded in water's natural processes, enabling continuous power generation. These technologies demonstrate promising application potential across various domains. This Review particularly emphasizes Bio-HEG applications in self-powered sensing, sustainable energy supply and environmental engineering, encompassing health monitoring, distributed energy systems, portable devices and environmental applications.

### Self-powered sensing

**Figure 5.**
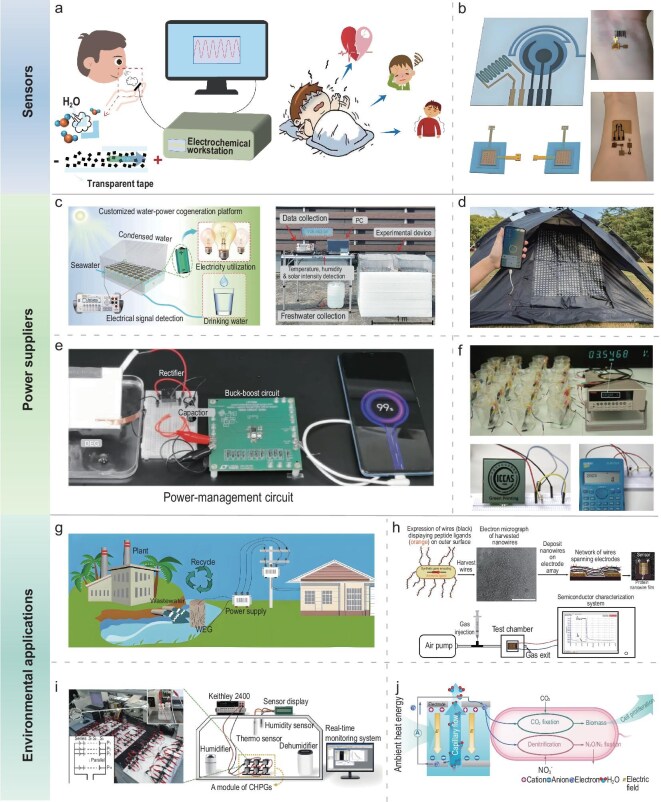
Applications and advancements of Bio-HEG. (a) Schematic diagram of breathing-monitoring processes [29]. Copyright 2023, Elsevier. (b) Illustration of connecting biofilm devices to wearable sensors for wearable power supply. (Right) Actual photographs showing (top) a skin-wearable sensor powered by a single biofilm device and (bottom) an electrochemical glucose sensor powered by three biofilm devices [14]. Copyright 2022, Springer Nature. (c) A self-designed outdoor freshwater-electricity cogeneration system [64]. Copyright 2024, Wiley-VCH GmbH. (d) Outdoor tent windows equipped with 8 × 240 series-parallel Mac fabric panels (front view), directly powering a mobile phone [90]. Copyright 2024, American Association for the Advancement of Science. (e) Photograph of a working DEG combined with a power management circuit charging a smartphone. Before charging the smartphone, the circuit is pre-charged by the DEG [92]. Copyright 2022, Elsevier. (f) Photographs of 24 sealed hydrovoltaic devices connected in series, powering small electronic devices [93]. Copyright 2024, Springer Nature. (g) A fully wood-based WEG device harvesting electrical energy from discharged alkaline wastewater for utilization [21]. Copyright 2024, American Chemical Society. (h) Schematic diagram of a protein nanowire-based sensor connected to a semiconductor characterization system and placed inside a custom airtight test chamber [94]. Copyright 2023, Elsevier. (i) Three parallel-connected MEG devices and their integration with a water-splitting hydrogen production device [47]. Copyright 2024, Elsevier. (j) Diagram of the metabolic mechanism of *R. palustris* for utilizing WEG [12]. Copyright 2024, Springer Nature. CHPG, cellulose sponge-based hydrovoltaic power generator.

Bio-HEG enables self-powered sensing by converting ambient humidity into electricity, eliminating the need for external power. This makes it ideal for health monitoring, such as tracking respiratory patterns, skin moisture and sweat biomarkers. For instance, in 2023, Fang *et al.* developed a respiratory monitor using carbonized rice husk (Fig. 5a) [29]. When placed about 10 cm from the nose, breathing caused a distinct humidity gradient and voltage output between covered and uncovered membrane regions. The device generated 30–50 mV from breath humidity, allowing stable detection over 205 cycles. Similarly, human skin produces humidity fluctuations from sweat and evaporation, which humidity sensors detect, generating current for touch-sensitive operation. In 2022, Yao *et al.* used microbial biofilms to harvest skin moisture, powering wearable devices for over 18 h. The breathable, polydimethylsiloxane (PDMS)-sealed design maintained adhesion and stability, enabling continuous monitoring of pulse, respiration and sweat glucose levels [14](Fig. 5b). These devices, complementary to Bio-HEG, offer sustainable power solutions for wearable sensing electronics.

### Sustainable energy supply

Bio-HEG demonstrates significant potential for sustainable energy supply, particularly in the realms of distributed energy systems and low-power devices through scalable designs. Inspired by the structural characteristics of lotus stems and leaves, and forest transpiration mechanisms, Tang *et al.* developed a WEG device with scalable and highly efficient performance [64]. By integrating a MEG device with a water treatment system, this technology achieved dual collection of electricity and freshwater. Using a chitosan-based hydrogel fabricated via liquid nitrogen-assisted templating, the system achieved dual electricity generation (45.6 µW·cm^−2^) and freshwater production (2.0 L·m^−2^·h^−1^), with a record 105 V output (Fig. 5c). This integrated approach addresses energy–water challenges, enabling round-the-clock operation through thermal storage. Wang *et al.* developed a plant-inspired fabric generator that produced 0.85 V for a month via moisture cycling [90]. Scalable configurations (500 × 300 units) deliver 350 V/33.76 mA, enabling off-grid mobile charging (Fig. 5d). Guo *et al.* optimized droplet energy harvesting through grounded capacitance analysis, achieving smartphone charging capability [92] (Fig. 5e). Song *et al.* demonstrated a sealed hydrovoltaic battery system maintaining 3.55 V output for >160 h via autonomous water circulation, powering LCDs and calculators [93] (Fig. 5f).

### Environmental engineering applications

Bio-HEG demonstrates significant potential in environmental engineering, particularly in wastewater treatment and sustainable energy production. Tian *et al.* developed a wood-based WEG that generates 1.1 V (*V*_oc_) and 320 µA (*I*_sc_) and, in alkaline wastewater, achieving a power output of 6.75 µW·cm^−2^ (Fig. 5g) [21]. Utilizing readily available water sources like rainwater and dew, WEG devices efficiently convert ion concentration gradients into electrical energy, exhibiting superior performance in alkaline wastewater compared to deionized water. In environmental monitoring, microbial protein nanowire sensors have expanded Bio-HEG applications. Lekbach *et al.* integrated peptide-functionalized nanowires into gas sensors for detecting ammonia and acetic acid, key biomarkers for kidney disease and asthma. These sensors enhanced sensitivity by approximately 100-fold for ammonia and 4-fold for acetic acid (Fig. 5h) [94]. Recent advances include Jeong *et al.*’s cellulose sponge-based MEG, which produced 0.47 V and 477 µA under ambient conditions (25°C, 45%–50% RH) [47]. By configuring six series and three parallel devices, they achieved a stable hydrogen production rate of 81 µmol·h^−1^ (Fig. 5i). In the same year (2024), Zhou *et al.* demonstrated that hydrovoltaic electrons generated through natural water evaporation could promote microbial growth while facilitating environmental remediation processes. The hydrogen ions produced during evaporation participated in cellular carbon fixation and reduction reactions, including nitrate conversion and carbon fixation (Fig. 5j) [12].

These developments highlight Bio-HEG's dual functionality in energy harvesting and environmental protection, showcasing its versatility in addressing contemporary ecological challenges.

## COMPARATIVE ANALYSIS OF NON-LIVING AND LIVING BIO-HEG

In the preceding sections, we systematically elucidated the fundamental mechanisms, materials and research progress of non-living and living Bio-HEG. This section compares the distinct characteristics of non-living and living Bio-HEG systems in material properties, scalability and applications.

### Material properties

Compared with non-living hydrovoltaics that primarily rely on static interfacial effects, living hydrovoltaics demonstrate superior sustained energy supply capabilities through dynamic biological regulation mechanisms (referring to the real-time regulatory capabilities, environmental adaptability and self-repair abilities inherent in the organisms themselves within living bio-hydrovoltaic systems, e.g. metabolic control, photosynthesis–respiration synergy). Although living hydrovoltaics may exhibit lower energy conversion efficiency during initial research stages, their multi-source coupling approach enables more stable energy output. For instance, plants can convert solar energy into chemical energy through photosynthesis and release electrical energy during transpiration, achieving dual solar/water energy utilization. Photosynthetic microorganisms harness light energy through photosynthesis, converting it to chemical energy. These organisms then generate electricity via bioelectrochemical processes, achieving relatively high conversion efficiency [11,18,71]. Biotechnological advances like genetic engineering may push living systems beyond non-living efficiency limits. Particularly in dynamic environments (such as fluctuating light intensity and temperature variations), living hydrovoltaics demonstrate remarkable environmental adaptability and metabolic regulation mechanisms, maintaining stable performance during long-term operation. Living hydrovoltaic systems can self-optimize and maintain dynamic equilibrium, unlike structurally fixed non-living materials. This adaptability delivers superior performance and higher engineering value in complex environments.

**Figure 6.**
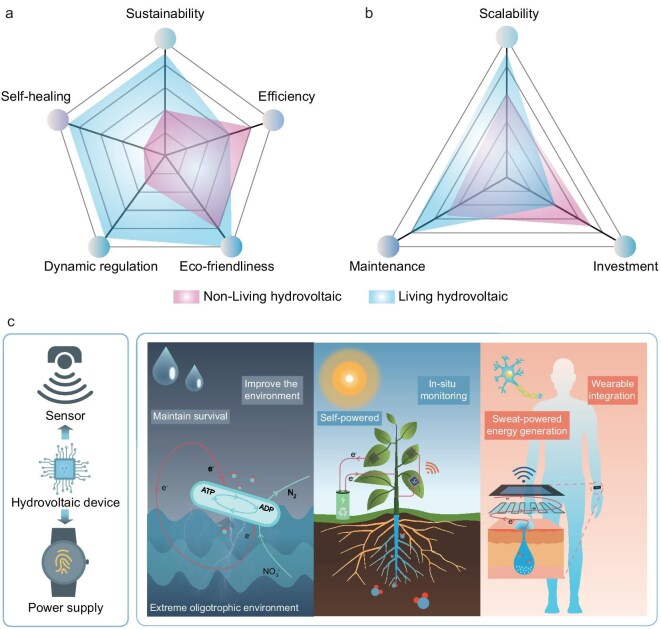
Comparison of living and non-living bio-hydrovoltaic systems. (a) A comparison of material properties (sustainability, efficiency, eco-friendliness, dynamic responsiveness, dynamic regulation and self-healing). (b) A comparison of sustainable expansion potential. (c) A comparison of application scenarios.

Both material types are inherently eco-friendly, as they are derived from renewable natural resources. Unlike many synthetic abiotic materials, these biomaterials are biodegradable and align with principles of green and sustainable development. Living systems offer additional environmental benefits, for example electricity generation using living plants does not require large-scale destruction or modification (Fig. 6a) [11].

### Scalability

Compared to the advantages of non-living hydrovoltaics in achieving scalable integration through modular production, living hydrovoltaics exhibit greater sustainable expansion potential through synthetic biology and microfluidic technologies. Living hydrovoltaic technology can significantly reduce long-term operational and maintenance costs through closed-loop nutrient cycling design. For instance, microbial electrochemical systems can treat wastewater while recovering organic matter for biofuel production. This approach enables multi-level resource recycling [95]. This self-sustaining metabolic network not only enhances energy conversion efficiency but also concurrently fulfills environmental remediation functions, offering both ecological benefits and economic advantages in large-scale applications, thereby promoting sustainable development (Fig. 6b).

### Application scenarios

Non-living hydrovoltaics possess technical maturity advantages in short-term, controlled scenarios (Fig. 6c, left), such as powering small electronics like smartwatches or sensors, or serving as micro-sensors for monitoring environmental temperature, humidity or human health conditions. In contrast, living hydrovoltaics demonstrate greater potential in long-term, complex and extreme environments through dynamic adaptation and self-healing mechanisms (Fig. 6c, right). For instance, in remote regions, this technology can enable outdoor environmental monitoring, including air pollution detection, soil quality assessment and even real-time feedback on plant health status. Moreover, in extremely oligotrophic environments, microorganisms can maintain physiological activities and generate energy through hydrovoltaic electrons.

Notably, there exists a natural compatibility between the distributed characteristics of living systems and decentralized energy network architectures. Such systems can form dynamic complementarity with other renewable energy sources (such as solar and wind energy), achieving autonomous supply–demand balance in micro-grids through localized energy-information collaborative regulation, thereby providing a unique technological pathway for constructing novel energy ecosystems.

## CONCLUSIONS AND STRATEGIC OUTLOOK

This review offers a comprehensive overview of bio-hydrovoltaic energy generation mechanisms, highlighting recent progress, performance characteristics and potential applications of both living and non-living hydrovoltaic systems. Although their power output is generally lower than that of carbon-based inorganic nanomaterial devices, bio-hydrovoltaics have advanced rapidly in recent years, with steady improvements in performance.

**Figure 7.**
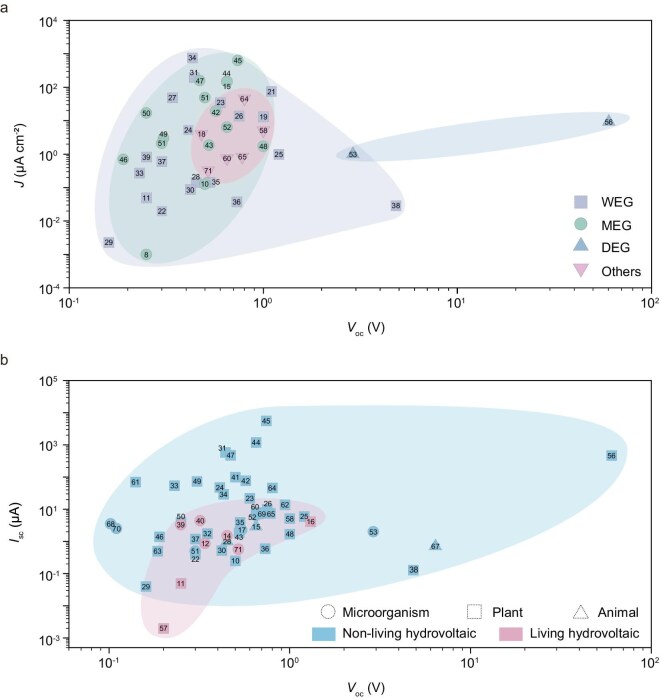
Comparison of the electrical performance of Bio-HEG devices. (a) Performance comparison between WEG, MEG, DEG and others. (b) Performance comparison between living and non-living Bio-HEG devices.

Based on current data, bio-WEG has been the most extensively studied, followed by MEG and DEG. In terms of power generation performance, as shown in Fig. 7a, WEG achieves higher voltage than MEG, while DEG generates the highest pulse voltages (bio-based systems only). Future research should focus on exploring the combination of multiple mechanisms to fully harness their respective advantages, thereby achieving more efficient and sustainable hydrovoltaic systems. This review classified hydrovoltaic material sources into microbial-, plant- and animal-based categories, with emphasis on their roles in living and non-living Bio-HEG devices. Among these, plant-based hydrovoltaic materials—especially wood and biochar—garnered the most attention due to their relatively higher open-circuit voltages and short-circuit currents. While microbial systems lag in output, they show strong potential in environmental applications. Research on animal-based materials remains limited, mostly focusing on biomimetic designs, with living animal-based bio-hydrovoltaics being almost non-existent.

Currently, research is predominantly focused on non-living Bio-HEGs, which deliver higher electrical performance (Fig. 7b). Nevertheless, living bio-hydrovoltaics, by integrating living biological functions, address long-term, complex and extreme environmental energy supply through dynamic adaptation and self-healing mechanisms. In this sense, living bio-hydrovoltaic technology possesses unique advantages and broader exploration potential.

As we consider future developments, bio-hydrovoltaic technology holds great promise for next-generation sustainable energy. Future development will be closely tied to advances in interdisciplinary strategies aimed at enhancing performance and exploring emerging research directions—particularly the nascent field of living hydrovoltaics.

### Strategies for enhancing bio-hydrovoltaics performance

To promote the advancement of bio-hydrovoltaic technology and improve its performance, future strategies may integrate methods from the following interdisciplinary fields.

#### Molecular dynamics simulations and machine learning

Bio-hydrovoltaic systems inherently involve multiphysics coupling at the molecular level. Molecular dynamics (MD) simulations can elucidate charge transport and ion migration mechanisms at the atomic scale, providing insights into interfacial dynamics and energy conversion processes at the water–material interface. Coupling MD with machine learning—e.g. convolutional neural networks (CNNs) for structure recognition or recurrent neural networks (RNNs) for signal analysis—these methods enable the prediction of structure–performance relationships and guide material optimization [96,97]. When integrated with experimental data, this approach can inform molecular-level design, such as surface functionalization and alignment, to enhance efficiency and stability. This synergy provides a powerful framework for translating lab research into practical applications.

#### Micro-/nano-fabrication and device integration

Non-living biomaterials offer inherent biocompatibility, making them ideal for flexible and miniaturized electronics. Advanced micro-/nano-fabrication enables precise integration of hydrovoltaic devices with sensors and processors on microchips, enhancing performance while reducing volume and weight. This facilitates the development of compact, efficient power systems for the Internet of Things (IoT) and wearable electronics. Moreover, their flexibility allows adaptation to dynamic human motion and environmental changes, providing lightweight and sustainable energy solutions for next-generation intelligent electronics.

#### Synthetic biology and gene engineering

Leveraging synthetic biology and gene-editing technologies provides innovative pathways for bio-hydrovoltaic technology development by precisely regulating microbial metabolic pathways and enzyme molecular structures [98,99]. The core challenge facing bio-hydrovoltaic technology is improving energy conversion efficiency and system stability, for which synthetic biology offers a systematic solution. Directional reconstruction of microbial metabolic pathways can optimize charge transfer mechanisms, enhance energy conversion efficiency of biologically living materials at water–material interfaces, and achieve precise performance regulation of materials. Constructing engineered cell libraries and enzyme element libraries not only enables rapid assembly and iteration of functional modules but also provides a flexible technical platform for developing high-stability hydrovoltaic systems, significantly shortening material optimization research and development cycles. More critically, by integrating synthetic ecology principles and regulating species composition and metabolic networks of biological communities, researchers can develop symbiotic systems adapted to specific environments, substantially improving the practicality and adaptability of living Bio-HEG devices across diverse natural environments [100]. This approach transcends traditional biological material performance bottlenecks, offering a novel theoretical framework and technological pathway for interdisciplinary bio-hydrovoltaic technology development. It transforms hydrovoltaic technology from single material optimization to systematic biological functional design, driving its evolution towards more efficient and intelligent directions and opening broad prospects for sustainable energy technology innovation.

**Figure 8.**
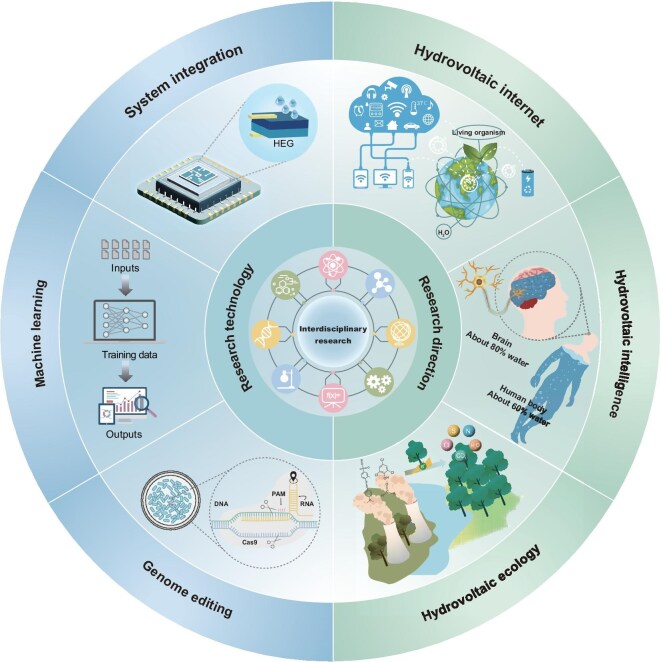
Future development of Bio-HEG systems. PAM, protospacer adjacent motif.

Together, these strategies chart a course toward efficient, durable and adaptable bio-hydrovoltaics—especially living systems. With continued innovation, the field will evolve along three directions (hydrovoltaic internet, hydrovoltaic intelligence and hydrovoltaic ecology) to build a highly efficient, intelligent and ecologically sustainable energy technology framework (Fig. 8).

### Future research directions for bio-hydrovoltaics

#### Hydrovoltaic internet

Bio-hydrovoltaic technology opens novel pathways for constructing next-generation distributed energy systems. First, by utilizing miniaturized Bio-HEG devices, people can efficiently capture low-grade energy from natural water-cycle processes (e.g. evaporation or moisture absorption), effectively compensating for existing distributed energy networks’ limitations in nanoenergy harvesting and dynamic power supply. Biomass materials, with their biodegradability, ecological compatibility and dynamic adaptability, provide more sustainable and flexible solutions for wearable devices and extreme environments. Notably, living hydrovoltaic technologies possess unique advantages, such as electricity generation induced by ubiquitous plant transpiration, which represents a natural distributed energy and self-powered sensing system. Through deep integration with intelligent devices and the internet, this distributed hydrovoltaic energy can further catalyze a novel ‘hydrovoltaic internet,’ utilizing IoT protocols to achieve energy scheduling and system efficiency optimization between devices. The resulting resilient energy matrix can not only flexibly adapt to remote off-grid regions but also serve energy demands across diverse scenarios like high-density urban clusters, significantly enhancing the future energy system's resilience and sustainability.

#### Hydrovoltaic intelligence

##### Adaptive and intelligent hydrovoltaic systems.

Due to the comprehensive advantages of bio-hydrovoltaic technology in environmental adaptability, sustainability and biocompatibility, future wearable devices can be designed with integrated self-powered and multi-modal sensing capabilities. For instance, future intelligent agricultural systems could employ bio-hydrovoltaic self-powered sensors for real-time monitoring of nutrients and moisture in soil. Integration with artificial intelligence and neuromorphic systems could enable precision agriculture via data-driven irrigation strategies. Furthermore, through fine-tuning of non-living material physicochemical properties, device adaptability and durability in complex environments can be effectively enhanced, thereby ensuring stable output and meeting multi-scenario requirements. Notably, the dynamic responsiveness of living Bio-HEG systems makes them particularly suitable for constructing intelligent and adaptive systems, capable of self-regulation and performance optimization.

##### Intelligent biomedical applications.

In the future, bio-hydrovoltaic technology will demonstrate extensive exploration potential in the biomedical domain. Ionic activities in the brain are closely related to water regulation, and hydrovoltaic technology is precisely built upon in-depth research into the interaction mechanisms of water and solids at quantum mechanical, atomic and biomolecular scales. The commonalities in their mechanisms and motion patterns not only provide novel insights for fundamental neuroscience research but can also be applied to cutting-edge fields such as artificial intelligence neural network systems and medical diagnostic biosensors.

For instance, in addressing neurological disorders like epilepsy, the potential to use hydrovoltaic technology's electrical signals for real-time reverse discharge regulation of abnormal brain electrical activity could offer innovative therapeutic approaches. By exploring the hydrovoltaic effects associated with blood flow in animal (human) bodies, researchers can design novel completely self-powered implantable medical devices such as cardiac pacemakers, biodegradable implantable medical devices or medical diagnostic biosensors. This approach opens new possibilities for disease monitoring and biological energy utilization, thereby promoting deep integration and innovation between hydrovoltaics and biomedicine.

#### Hydrovoltaic ecology

As a zero-carbon emission and negative thermal footprint technology, hydrovoltaic technology generates electricity without greenhouse gas emissions and offers innovative pathways to achieve carbon neutrality goals [3,4]. The essence of hydrovoltaic ecology lies in establishing a self-sustaining, decentralized and green sustainable energy ecosystem. By integrating hydrovoltaics with existing renewables like solar and wind, robust multi-energy systems can be developed to deliver decentralized energy solutions for remote regions, smart cities and sustainable agriculture. Such systems will exhibit unique advantages in environmental monitoring, dynamically sensing changes in temperature, humidity and pollutants. Moreover, the energy synergistic effects among animals, plants and microorganisms within the environment will promote the production of renewable energy, achieving a deep integration of natural ecology and energy production.

Future research will extend hydrovoltaic ecology to ecological restoration, including pollutant detection and degradation. This includes constructing electrons or free radicals generated during the bio-hydrovoltaic process to degrade or eliminate pollutants and pathogenic microorganisms. This research direction will aid ecological restoration and sustainable development, providing greater possibilities for exploring interactions between future energy and ecosystems.

In summary, current Bio-HEG systems still face numerous challenges, including insufficient material stability, low energy conversion efficiency, limited system durability and high costs of large-scale production. Additionally, the issues of biosafety, seasonal variations in power output, and low performance in living hydrovoltaic devices also require significant attention. These deficiencies may limit their adoption in practical applications. To ensure that large-scale applications are environmentally harmless, thorough toxicity and biosafety assessments must be conducted, and ecological impacts should be considered in material selection and system design. To accelerate progress in these areas, interdisciplinary collaboration among biologists, engineers and materials scientists will be essential. Through collective efforts, these experts can address the technological, environmental and economic challenges facing bio-hydrovoltaic technology, promoting the widespread application of this promising technology. Specifically, future research should focus on enhancing material stability, optimizing energy conversion efficiency, improving system durability and reducing costs of large-scale production, while also paying special attention to how to improve device output performance and long-term stability, thereby making bio-hydrovoltaic systems feasible for large-scale commercial deployment and providing practical solutions for sustainable energy development.
